# Land Subsidence and Its Relations with Sinkhole Activity in Karapınar Region, Turkey: A Multi-Sensor InSAR Time Series Study

**DOI:** 10.3390/s21030774

**Published:** 2021-01-24

**Authors:** Osman Orhan, Talib Oliver-Cabrera, Shimon Wdowinski, Sefa Yalvac, Murat Yakar

**Affiliations:** 1 Department of Geomatics Engineering, Engineering Faculty, Mersin University, 33100 Mersin, Turkey; osmanorhan@mersin.edu.tr (O.O.); myakar@mersin.edu.tr (M.Y.); 2 Institute of Environment, Department of Earth and Environment, Florida International University, Miami, FL 33199, USA; toliver@fiu.edu; 3 Department of Geomatics Engineering, Engineering Faculty, Gumushane University, 29000 Gumushane, Turkey; sefayalvac@gmail.com

**Keywords:** InSAR, subsidence, sinkhole, COSMO-SkyMed, sentinel 1, groundwater overpumping

## Abstract

The Karapinar basin, located in the Central Anatolian part of Turkey, is subjected to land subsidence and sinkhole activity due to extensive groundwater withdrawal that began in the early 2000s. In this study, we use Interferometric Synthetic Aperture Radar (InSAR), Global Navigation Satellite System (GNSS), and groundwater level data to monitor and better understand the relations between groundwater extraction, land subsidence, and sinkhole formation in the Karapinar basin. The main observations used in the study are InSAR-derived subsidence velocity maps calculated from both Sentinel-1 (2014–2018) and COSMO-SkyMed (2016–2017) SAR data. Our analysis reveals broad areas of subsidence with rates exceeding 70 mm/yr. The InSAR-derived subsidence was compared with GNSS data acquired by a continuously operating GNSS station located in the study area, which show a similar rate of subsidence. The temporal characteristic of both InSAR and GNSS time series indicate a long-term subsidence signal superimposed by seasonal variability, which follows the overall groundwater level changes, with over 80% cross-correlation consistency. Our results also indicate that sinkhole activity is limited to slow subsidence areas, reflecting strong cohesion of near-surface rock layers that resist subsidence but yield to collapse in response to aquifer system deformation induced by groundwater extraction.

## 1. Introduction

The main reason for the increasing occurrence of non-tectonic geological hazards (subsidence and sinkholes) is anthropogenic usage of water resources due to rapid increase of the world population and the corresponding need to accommodate and meet daily needs [1,2]. Land subsidence occurs in built and agricultural areas and is related to groundwater extraction due to the compression of overdraft aquifer systems [3,4]. Sinkhole formation occurs in areas where bedrock consists of highly soluble calcium carbonate or evaporitic rocks, such as limestone and rock salt. Increased land subsidence and sinkhole occurrence, which previously were not perceived as a threat in areas of low population density, limited agricultural activities, and restricted industrial areas, have now posed a greater danger and risk to human life [5]. Sinkholes can cause severe financial losses, especially in built environments, and may unfortunately result in human loss. Therefore, monitoring the spatial and temporal distribution of land subsidence and the sinkhole activity is needed for disaster prevention and sustainable development of agriculture and urban areas.

In recent years, land subsidence and sinkhole activity have been detected and monitored using Interferometric Synthetic Aperture Radar (InSAR) observations [6,7,8,9,10,11,12,13,14,15,16,17,18]. InSAR is a microwave remote sensing technique that provides information on spatial and temporal surface deformations using a phase difference between two SAR acquisitions in the line of sight (LOS) between the satellite and the surface. Differential InSAR (DInSAR), which is the classical InSAR method, detects surface displacement between two SAR acquisition times. However, this technique restricts the monitoring of the temporal change in surface deformation and has a few disadvantages that limit its usage [19,20,21,22,23]. These disadvantages consist of errors such as perpendicular baseline, spatial–temporal decorrelation, digital elevation model error, and tropospheric delay. In order to reduce these difficulties and monitor the temporality of deformation changes, InSAR time series techniques were developed, such as Persistent Scattering Interferometry (PSI) [19] and Small BAseline Subset (SBAS) [24].

SAR technology and SAR satellite missions have been improved over the years. In 2014, the European Space Agency (ESA) Sentinel-1 launched the Sentinel-1 satellite mission, which operates a C-band radar (5.56 cm wavelength) with 6–12 days repeat orbit. Sentinel-1 data has been widely used for monitoring land subsidence in areas prone to sinkhole formation worldwide, including Texas [25], Iran [26], and Western Central Florida [27]. Sinkhole-induced deformation was also detected by X-band (3.1 cm wavelength) COnstellation of small Satellites for the Mediterranean basin Observation (COSMO SkyMed) satellite constellation and the TerraSAR-X satellite [28,29,30]. The precursory deformation, which are harbingers of sinkhole formations, were detected along the Dead Sea (DS) shorelines in Israel using InSAR measurements by using COSMO-SkyMed satellites, by [28,29]. These studies show that in some cases, InSAR observations used to determine the progress of sinkhole induced deformation prior to the sinkhole’s collapse and, hence, can be used to minimize damage to people and property.

**Figure 1:**
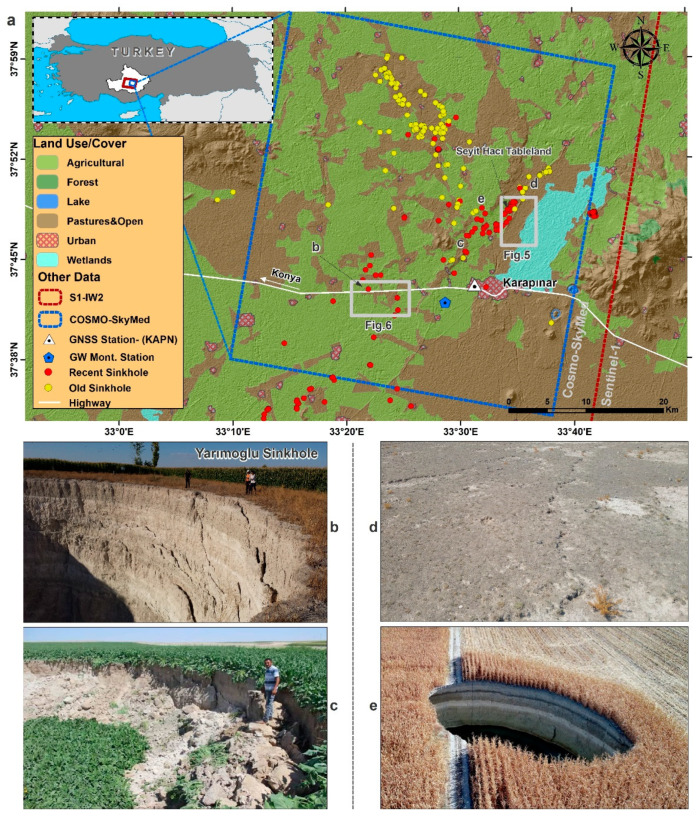
(**a**) Location of the study area on a Land Use/Land Cover map. Yellow and red circles mark the locations of old and recent sinkholes, respectively. Blue and Red frames show the footprints of the COSMO-SkyMed and Sentinel-1, respectively. Grey frames mark the locations of Figures 5 and 6. (**b**) Yarım oğlu Sinkhole. (**c**) A sinkhole formed in the beet field on 23 August 2016. (**d**) Some surface fissures. (**e**) A sinkhole formed in the cornfield in September 2018.

In Turkey, the Karapınar region located in Konya Closed Basin, has been affected by land subsidence and sinkhole activity (Figure 1). The area is an essential agricultural basin of the country. For centuries, agricultural activity in the basin was conducted by dry farming. However, since the 2000s, irrigated farming areas have increasingly become dependent on groundwater usage in and around Karapınar [31]. The increased usage of groundwater in Karapınar and its vicinity increased land subsidence and sinkhole formations in the region over the past decade [32,33,34]. A previous InSAR study of land subsidence based on Envisat data acquired during the years 2002–2010 revealed the occurrence of land subsidence over wide areas at a maximum rate of 15 mm/yr [35]. Sinkhole studies in the Karapinar area were based on ground observations, such as the study of [36], which detected a total of 102 sinkholes. More recently, Reference [37] reported that 332 sinkholes were formed in the region. The increased sinkhole formation reflects population growth, rapid economic development, and the sharp increase in agricultural groundwater usage.

In this study, we expand the InSAR investigation of land subsidence in Karapınar region [35] using different data types that were acquired more recently. We analyze both Sentinel-1 (2014–2018) and COSMO SkyMed (2016–2017) SAR data, which were processed using the SBAS technique. The study is also based on Global Navigation Satellite System (GNSS) observations and monthly groundwater level data acquired in the study area. All three data types allow us to detect spatial and temporal patterns of land subsidence and obtain a better understanding of the relations between groundwater extraction, land subsidence, and sinkhole formation in the Karapinar basin.

## 2. Study Area

The Karapınar region, covering 2940 km^2^, is a sub-basin located within the semi-arid Konya Closed Basin (KCB) in the Central Anatolian Plateau, at an elevation of 980–1080 m (Figure 1). Most of the Karapınar region is characterized by an arid climate. There is also a desert area in the southern parts of the region characterized by a low precipitation level, annual mean of 265 mm/yr (based on rain record from 1964–2015), which is the lowest in Turkey. Moreover, the evaporation in the region is generally higher than the precipitation [38]. The Karapinar area has been used for agricultural production for centuries, mostly by dry farming (non-irrigation). However, since the early 2000s, irrigated agrarian activities, mainly for sugar beet and corn, which rely on groundwater extraction, have increased the agricultural productivity of the area [33].

During the last two decades, changes in agriculture patterns induced various environmental problems, including land subsidence, sinkholes formation, and surface fissures in the region. For example, there were 332 sinkholes in Karapınar and its surroundings by 2020 [37]. These environmental problems arise due to groundwater over-pumping, which is needed for irrigated agriculture [33]. Previous studies estimated that irrigation has been conducted using roughly 92,000 groundwater wells, including 66,000 that have been used illegally, throughout the KCB [35,39]. We estimated a total of 6000 wells in the Karapinar sub-basin alone.

### Geology

**Figure 2:**
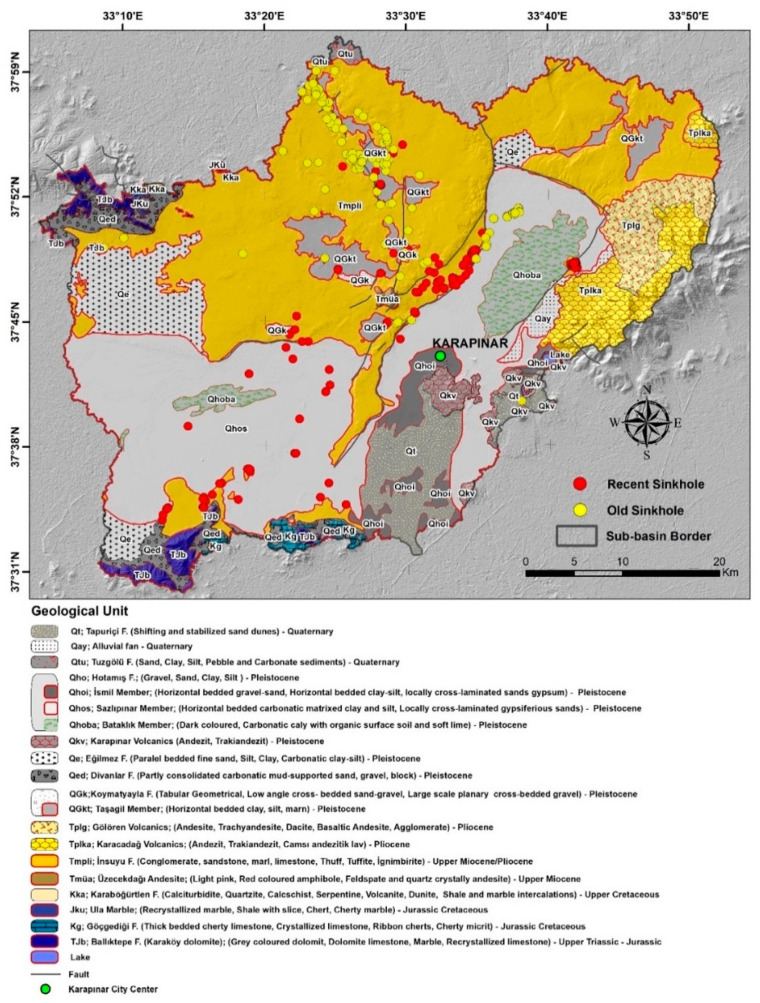
Geology map (modified from [41]) with a distribution of 332 sinkholes in the study area. The grey line marks a fault. The yellow and red circles mark old and recent sinkholes, respectively.

The study area can be divided into three main geological units, including Obruk plateau in the northern parts, Karapınar plain in regions extending from southwest to the northeast, and volcanic field in the southeastern section (Figure 2). Obruk plateau is generally covered with Miocene–Pliocene conglomerate, sandstone, marl, limestone, tuff, and evaporites. The Karapinar plain consists of Pleistocene aged sediments, mainly clay, silt, and sandstone. In addition, some parts of the plain are covered by Quaternary alluvial fan deposits. Volcanic mountains cones, maars, and other volcanic structures were formed during a Miocene–Quaternary volcanic phase and are visible in southeastern parts of the study area [38,40].

Karst features and land subsidence occur in the Insuyu and Hotamış formations (Tmpli and Qho geological units in Figure 2) and can be observed throughout the Karapınar Basin. The leading causes for karst formation are limestone dissolution in the subsurface, whereas the leading causes of land subsidence are the presence of fine-grained materials in the shallow subsurface [33].

## 3. Data and Data Processing

Our study of the Karapinar sub-basin relies on three data types, InSAR, continuous GNSS, and groundwater level. The InSAR data provide high spatial resolution (3–20 m) but moderate temporal resolution (12–16 days) observations of the study area. The continuous GNSS observations were acquired in a single site and provide high temporal resolution of ground movements, which are used to verify the InSAR observations. The groundwater level data were acquired in a single site with high temporal resolution. The groundwater level data were used to reveal the relationship between land subsidence and groundwater.

### 3.1. InSAR Data

Karapinar and its surroundings were investigated using two Synthetic Aperture Radar (SAR) datasets. The first dataset was acquired by the COSMO-SkyMed satellites in StripMap HIMAGE mode and the second dataset by the Sentinel-1 satellites in interferometric Wide (IW) swath mode. Our first dataset consists of 25 COSMO-SkyMed images, which were acquired between 16 February 2016 and 29 November 2017 with 3 m pixel resolution. This X-band (wavelength 3.1 cm) data were acquired in descending mode with VV polarization. The second SAR dataset consists of 87 Sentinel-1A images, which were acquired between 5 October 2014 and 6 March 2018 with 5 m × 20 m pixel resolution. This C-band (wavelength 5.6 cm) data were also acquired in descending mode with VV polarization. The footprints of the COSMO-SkyMed and Sentinel-1 satellites cover well the study area, as shown by the blue and white frames, respectively, in Figure 1.

### 3.2. InSAR Data Processing

Both datasets were processed using a two-step procedure. First, interferograms were generated using criteria as described below (Section 3.3), and then InSAR time series analyses were calculated using the SBAS technique to obtain velocity values. For Sentinel-1 data, we used the ROI_PAC software [42] to generate interferograms and The Miami INsar Time-series software in PYthon (MintPy) package [43] to calculate the time series. For COSMO-SkyMed data, we used the ENVI SARscape 5.5 (Exelis Visual Information Solutions, Boulder, CO, USA) software to generate interferograms and to calculate time series products.

All interferograms were calculated using the SRTM Global 1 arc second dataset [44,45] in order to remove the topographic component from the phase component. In addition, the multi-looking process was applied to all DInSAR results to reduce phase noise in both range and azimuth directions. The resulting ground resolution was 20 m for the COSMO-SkyMed and 100 m for the Sentinel-1 datasets. We also performed interferogram unwrapping and filtering to improve the signal-to-noise ratio [46]. Temporal coherence is considered as univariate exponential function of time, calculated by taking the random motion of scatterers in a resolution cell [47]. It ranges from 0, where there is no useful information, to 1, where there is no noise. A threshold for temporal coherence is used to select SAR pixels with reliable results. Therefore, in order to extract decorrelated pixels from SBAS results derived from COSMO-SkyMed and Sentinel-1 data, we selected only SAR pixels that exhibited a temporal coherence value larger than 0.7.

### 3.3. InSAR Time Series Analysis

**Figure 3:**
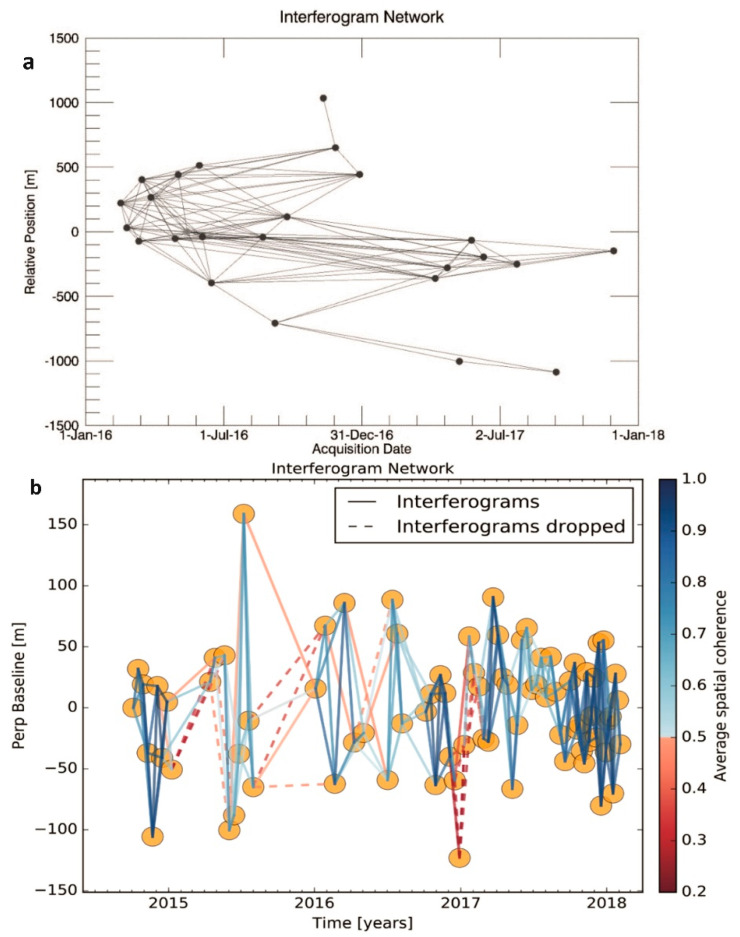
(**a**): Interferogram network configuration of COSMO SkyMED images processed by Small BAseline Subset (SBAS). (**b**): Interferogram network configuration of the Sentinel-1 images processed by SBAS.

We used the SBAS algorithm to calculate surface velocity with 25 COSMO-SkyMed images, which were acquired during a 2-month period (16 February 2016–29 November 2017). Our criteria for interferogram selection included (1) a maximum perpendicular baseline of 500 m and (2) a maximum temporal baseline of 400 days, which resulted in a total of 122 interferograms (Figure 3). The final displacement velocity map covering the entire Karapınar area had dimensions of approximately 40 × 40 km^2^.

Interferogram selection for the Sentinel-1 dataset, which includes 87 acquisitions, was based on a different strategy due to the orbital stability of Sentinel-1 along with small orbital baselines [48]. The interferogram network was constructed by connecting each SAR image with its three consecutive SAR images, which resulted in a total of 254 interferograms. The final displacement velocity map covering the Karapınar area had the dimensions of approximately 90 km (width) × 70 km (length).

One of the essential steps of this study is to investigate the accuracy of the time series obtained from InSAR data with the help of GNSS data located. In order to compare the deformation information collected from InSAR with GNSS, the deformations in the LOS direction that represent movements away from the satellite must first be converted into vertical deformation information. The time series of the vertical deformation can be obtained from the LOS measurements [49,50] as follows:

(1) $$disp{\;}_{vert}≅\frac{disp_{LOS}}{\cos\vartheta }$$

where $disp{\;}_{vert},\;disp_{LOS}\;$ and ϑ are vertical deformations, LOS deformation, and the sensor’s incidence angle, respectively. In this study, we used mean incidence angles of 37.30° and 33.80° for COSMO-SkyMed and Sentinel-1 data, respectively. This equation is based on the assumption that horizontal deformation in the study area is negligible.

### 3.4. GNSS Data Analysis

Continuous GNSS measurements in the study area were obtained by a single station (KAPN) of the Turkish National Permanent RTK Network (CORS-TR) (Figure 1), which has been operated by the General Directorate of Mapping (GDM) since 2008. We processed daily Receiver Independence Exchange (RINEX) observation files using GAMIT/GLOBK V10.70 software [51]. The analyses were carried out in two steps. In the first step, the relative coordinates were estimated based on the weighted least squares algorithm using the ionosphere-free linear combination (LC) of the phase observable by the GAMIT module. The orbital and clock parameters were obtained from International GNSS Service (IGS), and the minimum constraint (with respect to the ANKR site) procedure was used for ambiguity fixing in 5 cm for both horizontal and vertical directions. In the second step, the reference frame definition was performed for the daily solutions using the GLRED module. Then, a 7-parameter Helmert transformation was applied and its parameters were estimated utilizing 10 IGS stations (ANKR, ARUC, BSHM, HAMD, ISTA, MATE, NICO, ORID, TUBI, and ZECK) with coordinates and velocity defined in ITRF14.

### 3.5. Groundwater-Level Data

Groundwater data were obtained from a single well named 52258, which is located in the center of the study area (Figure 1). The well measurements were provided from the General Directorate of State Hydraulic Works and provide daily water level values for the time period of 2014–2018.

## 4. Results

**Figure 4:**
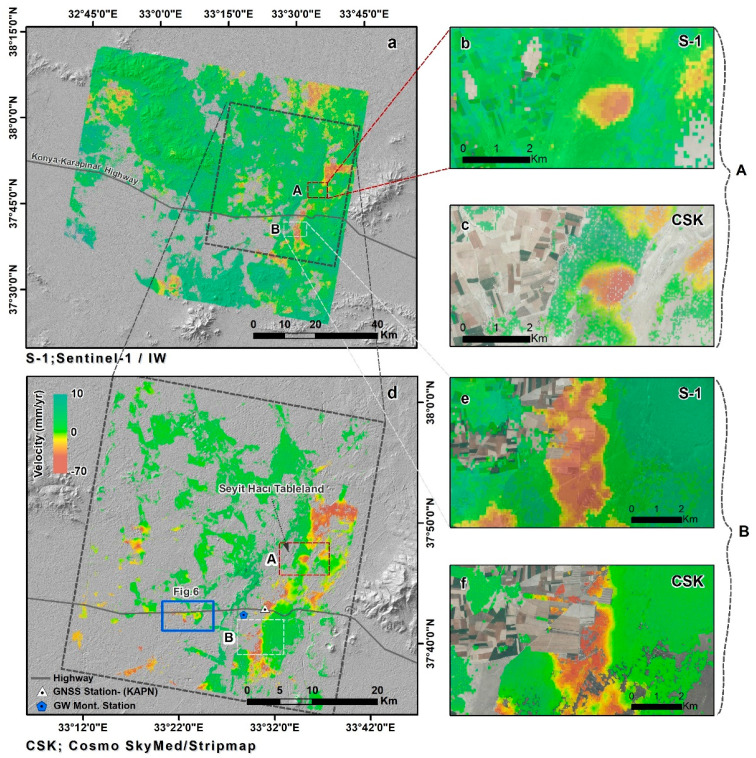
Velocity maps in line of sight (LOS) of the Karapınar study area derived from Sentinel-1 (**a**,**b**,**e**) and COSMO SkyMed (**d**,**c**,**f**) data. The velocity maps are laid over shaded relief maps derived from the SRTM dataset. The red and white dashed frames mark the locations of two zoom-in areas shown in (**b**,**c**,**e**,**f**). The triangle and blue pentagon mark the locations of the Global Navigation Satellite System (GNSS) stations and the groundwater well used in the study, respectively. Blue frames (in (**d**)) mark the locations of Figure 6.

InSAR time series analysis of the two datasets (COSMO-SkyMed and Sentinel-1) yielded similar results in terms of subsidence patterns and rates (Figure 4). Although the two datasets have different spatial coverage and coherence levels, both maps reveal an overall low subsidence rate throughout most of the study area (≤10 mm/yr; green in Figure 4), with patches of high subsidence rates (≤50 mm/yr; brown in Figure 4) mostly in the eastern part of the study area. A closer look at two regions with patches of high subsidence rate show very similar subsidence patterns and rates detected in both COSMO-SkyMed and Sentinel-1 velocity maps (Figure 4a,d). The two datasets yielded slightly different LOS velocity rates: COSMO-SkyMed velocity ranged between −70–10 mm/yr, and Sentinel-1’s velocity ranged between −60–10 mm/yr. These detected changes reflect different subsidence rates that occurred at different observation periods. The slightly higher rate measured by COSMO-SkyMed detected deformation between February 2016 and September 2017, whereas the slightly slower rate measured by Sentinel-1 detected subsidence during a longer period of 2014–2018. InSAR results are calculated and presented in LOS, which include both vertical and horizontal deformation components. Horizontal deformation in the Karapınar region is expected to be small, as the region is within the interior of the Anatolian Block far from seismically active faults. Thus, we assume that the entire LOS observed displacements represent vertical movements. Such assumption was successfully used by other studies of land subsidence and sinkhole activities [17,50]. Our assumption of dominant vertical movements is also supported by the comparison of InSAR data with vertical GNSS, as presented in the discussion.

**Figure 5:**
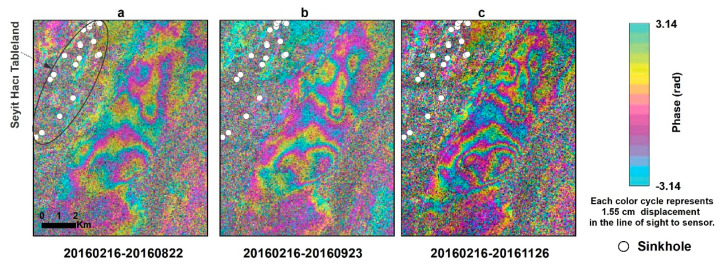
Land subsidence evolution of an area located north of the Karapinar (area A in Figure 4), as observed by COSMO SkyMed Interferograms. (**a**) Six-month interferogram showing 1–2 fringes of subsidence. (**b**) Seven-month interferogram showing 2–3 subsidence fringes. (**c**) Nine-month interferogram showing 3–4 subsidence fringes, mostly in the southern part of the subsiding area.

**Figure 6:**
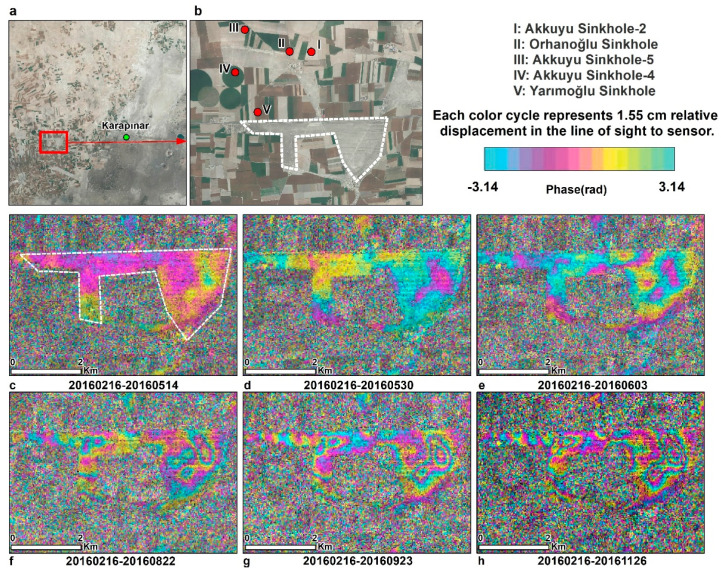
Land subsidence evolution of an area located east of the Karapinar, as observed by COSMO SkyMed Interferograms. (**a**) Orthophoto image showing the location of the subsiding area. (**b**) Zoom-in image of the subsiding area with sinkhole locations. (**c**,**d**) Three-month interferogram showing no subsidence. (**e**) Four-month interferogram showing the beginning of the subsidence. (**f**) Six-month interferogram showing 1–2 fringes of subsidence. (**g**) Seven-month interferogram showing 2–3 subsidence fringes. (**h**) Nine-month interferogram showing 3–4 subsidence fringes, mostly in the east part of the subsiding area.

Subsidence evolution over time can be detected using a series of interferograms. We present two examples of subsidence evolution using COSMO-SkyMed interferograms over time intervals of 3–9 months. We chose to use X-band COSMO-SkyMed interferograms rather than C-band Sentinel-1, because the shorter wavelength of X-band (3.1 cm) interferograms has higher detection capability than the C-band (5.6 cm) interferograms. The two study areas shown in Figure 1 are (1) the Seyithacı tableland, located in the north of Karapınar city center (Figure 5), and (2) a bare area, located in the southern part of the Karapınar-Konya highway, which is also located in the southern part of the Yarimoglu sinkhole (Figure 1b), which was formed in 2009 (Figure 6). These two selected areas present the clearest fringes in the entire study area (Figure 5 and Figure 6).

The subsidence evolution of the Seyithacı tableland is presented by a series of three interferograms spanning over a period of 3–9 months (Figure 5). All three interferograms share a common acquisition (20160216), which allows us to detect subsidence evolution with respect to the same initial time. The 6-month-long interferogram shows two epicenters of subsidence, one in the southern part of the area and the second in the northern part (Figure 5a). The maximum subsidence in this 6 month period is ~40 mm (2 fringes); each X-band LOS fringe translates to ~20 mm in vertical using Equation (1) with an incidence angle of 37.30° and LOS displacements of 15.5 mm, with an X-band of one fringe cycle. The deformation evolution indicates increased subsidence mostly in the southern section of the study area, in which maximum subsidence reached 40–50 mm (2–3 fringes; Figure 5b) after 7 months and 60–70 mm (3–4 fringes; Figure 5c) after 9 months. This example indicates that subsidence varies both spatially and temporally.

The subsidence pattern shows an overall increase in accumulated subsidence mainly in the southern part of the study area. The subsidence evolution also indicates an increased number of localized subsidence (closed fringes) from two locations after 6 months (Figure 5a) to four and possibly five locations after 9 months (Figure 5c). The subsidence rate also varies with time. The first two fringes (~40 mm) were developed during a 6 month period (February to August, 2016; Figure 5a), whereas the additional two fringes, from two to four fringes in Figure 5c, occurred within a 3 month period (August to November, 2016).

The subsidence evolution in the bare area located in the southern part of the Karapınar–Konya highway was studied using six interferograms spanning over a period of 3–9 months. As in the previous example, all interferograms share the same starting acquisition of 20160216. Initial subsidence of ½ fringe (~10 mm) can be detected after 3 months in the eastern side of the area (Figure 6d). The subsidence evolved in both eastern and western parts of the study area. The subsidence rate also varied with time. The subsidence during the first three months (February to May, 2016; Figure 6c,d) reached only ½ a fringe. However, the accumulated subsidence increased from ½ a fringe to 2 fringes (Figure 6f) within the next 3 months (June to August, 2016), and to 4 fringes (Figure 6h) after additional 6 months (June to November, 2016). This observed rate change suggests a seasonal subsidence pattern of limited subsidence in the spring (February to May) and fast subsidence in the summer and fall (June to November).

**Figure 7:**
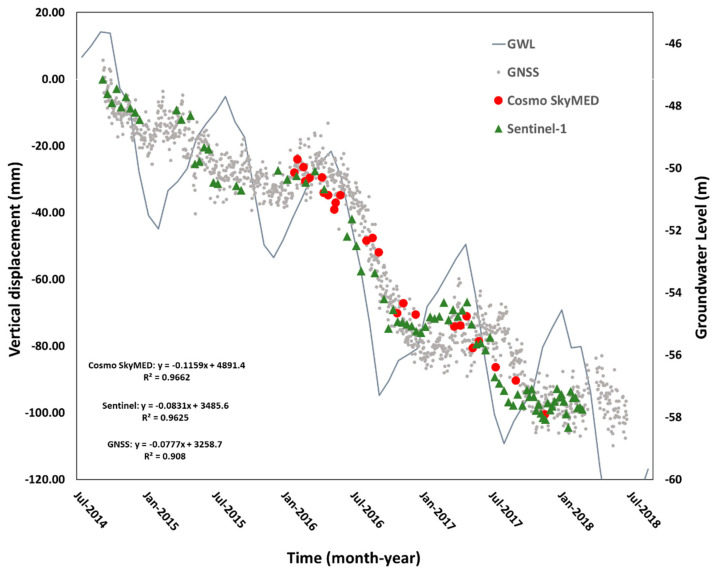
Vertical displacement time series at the KAPN GNSS station derived from GNSS, COSMO-SkyMed and Sentinel-1 observations. The figure also displays groundwater level measured at a well (well no: 52258), which is located 3 km southwest of the KAPN station.

High temporal resolution observations were obtained from the KAPN GNSS station and groundwater level measurements acquired in well number 52258. The GNSS station is located in the western outskirts of Karapinar, and the well is located 3 km west of the GNSS station in an open agricultural area (Figure 1 and Figure 4). The GNSS observations were compared with InSAR time series derived from the Sentinel-1 and COSMOS-SkyMed, which were projected from LOS to vertical. Time series of the vertical GNSS daily solutions for the same observation period of the Sentinel-1 data (May 2014–May 2018) revealed an accumulated subsidence of 100 mm, suggesting an average subsidence rate of 25 mm/yr. However, the time series also shows a seasonal subsidence pattern, in which the seasonal component varies from one year to another (Figure 7). The highest seasonal subsidence occurred in 2016–2017, whereas the lowest seasonal changes occurred at the beginning of the observation period, in 2014–2016. In the last two years of the observation period (2017–2018), the seasonal component was higher than at the beginning of the observations (2014–2016) but lower than in the 2016–2017 period.

The InSAR time series, which was projected from LOS to vertical, show a very similar seasonal subsidence pattern as the GNSS observations (Figure 7). We calculated the difference between the GNSS and the InSAR time series using Root Mean Square Error (RMSE) analysis. The RMSE between the GNSS and the Sentinel-1 observations is 7.13 mm and between the GNSS and the COSMO-SkyMed is 4.21 mm. These results suggest that the accuracy of the InSAR positioning is in the range of 4–7 mm.

The groundwater level time series indicates an overall decline in groundwater level with a strong seasonal component (grey line in Figure 7). The accumulated groundwater level during the four-year observations period is 12 m, suggesting an average water level drop of 3 m/yr. The ground level seasonal component varies from one year to another in the range of 3–7 m. The highest seasonal decline occurred in 2016–2017 (7 m), and the lowest decline occurred in 2015–2016 (3 m).

**Figure 8:**
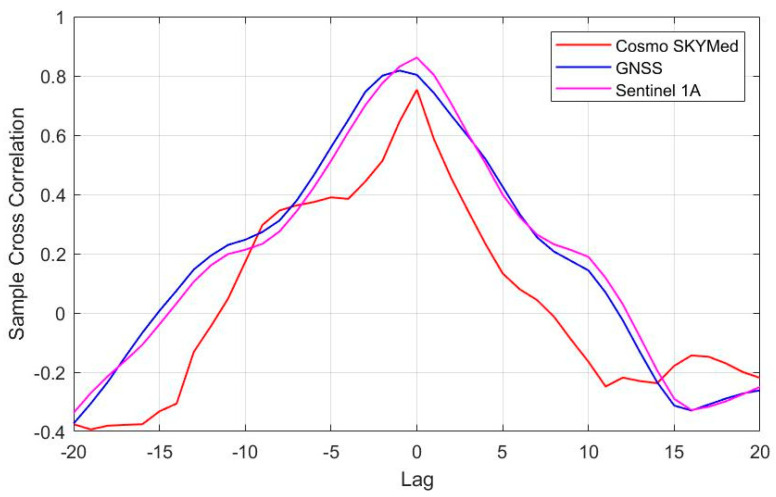
Cross-Correlation histogram between groundwater level and land subsidence derived from GNSS, COSMO-SkyMed, and Sentinel-1.

The vertical displacements obtained from GNSS, COSMO-SkyMed, Sentinel-1, and groundwater level observations show very similar temporal patterns of an overall decline with seasonal components. In order to quantify these relations, we conducted cross-correlation analyses between the four independent time series (GNSS, COSMO-SkyMed, Sentinel-1, and groundwater level). The cross-correlation analysis provides a comparison between two-time series and objectively finds how they match. In this analysis, the lag refers to time-shift between two series (vertical displacement and groundwater level). The cross-correlation analysis results revealed that there is no offset between groundwater level and land subsidence derived from Sentinel-1 and COSMO-SkyMed data. However, the analysis detected a month lag between the groundwater level and the land subsidence derived from GNSS (Figure 8). The cross-correlation coefficient between groundwater level data and vertical displacements obtained from GNSS, COSMO-SkyMed, and Sentinel-1 have been found 0.83, 0.78, and 0.88 respectively.

## 5. Discussion

Within the scope of this study, land subsidence due to groundwater over-pumping in the Karapınar region was determined by the InSAR time series using 25 COSMO-SkyMed images acquired between 2016–2017 and 87 Sentinel-1A images acquired between 2014–2018. The results reveal that the Karapınar region experienced significant land subsidence, reaching 60 mm/year from 2014 to 2018. A similar study conducted by [35] indicated that land subsidence rates in Karapınar during the years 2002–2010, as determined by Envisat data, was approximately 15 mm/yr. The increasing subsidence rate in recent years, compared to a decade ago, indicates an acceleration in the rate of subsidence, which we attribute to the increasing extraction of groundwater in the area. The main reason for this higher groundwater extraction is the gradual conversion of farming practices from non-irrigated agriculture in the region two decades ago to irrigated agriculture since 2010.

This study relies on two datasets, X-band COSMO-SkyMed and C-band Sentinel-1A, which detected similar rates and spatial patterns of subsidence (Figure 4). However, there are three main differences between subsidence maps calculated from the two datasets. First, the subsidence detected by the Sentinel-1A dataset covers a significantly larger area than COSMO-SkyMed, because the swath of the Sentinel-1 scene is 250 km wide, whereas the swath of COSMO-SkyMed is only 40 km. Second, the coverage of the Sentinel-1A dataset within the same frame as COSMO-SkyMed is more continuous, reflecting a better coherence of the C-band Sentinel-1A compared to the X-band COSMO-SkyMed data. The improved coherence of the Sentinel-1 data reflects the use of longer radar wavelength, but also the improved repeat orbits of the Sentinel-1 mission, which are constrained within the 150 m envelope compared to the >1000 m envelope of the COSMO-SkyMed mission (see perpendicular baselines in Figure 3). Third, the COSMO-SkyMed dataset had a slightly higher subsidence velocity value than those produced from Sentinel-1 data. The higher rate of the COSMO-SkyMed data was calculated from an only 19-month period, in which groundwater level drop was very rapid due to the excessive usage (Figure 7). Consequently, the subsidence velocity during the fast-dropping water level period (COSMO-SkyMed) is higher than the mean velocity during the longer period (Sentinel-1), which includes periods of fast and slow rates of water level drop.

The temporal subsidence pattern obtained from the GNSS, COSMO-SkyMed, and Sentinel-1 observations revealed a continuous subsidence with a significant seasonal component (Figure 7). The amplitude of the seasonal component was in the range of 10–30 mm, which varies from one year to another. The seasonal component was also noticeable in the COSMO-SkyMed interferogram time series showing a lower subsidence rate during the winter and spring months compared to a faster rate during the summer months (Figure 5 and Figure 6). The seasonal subsidence signal follows with a minimal lag in the observed seasonal component of groundwater level (Figure 7), suggesting a time-independent surface deformation response to seasonal water level changes. Similar seasonal subsidence behavior was observed in many locations, including Las Vegas [4,52], California [53], and Beijing [54]. The seasonal subsidence component reflects poroelastic deformation of the aquifer system in response to stresses induced by seasonal hydraulic head changes [55].

**Figure 9:**
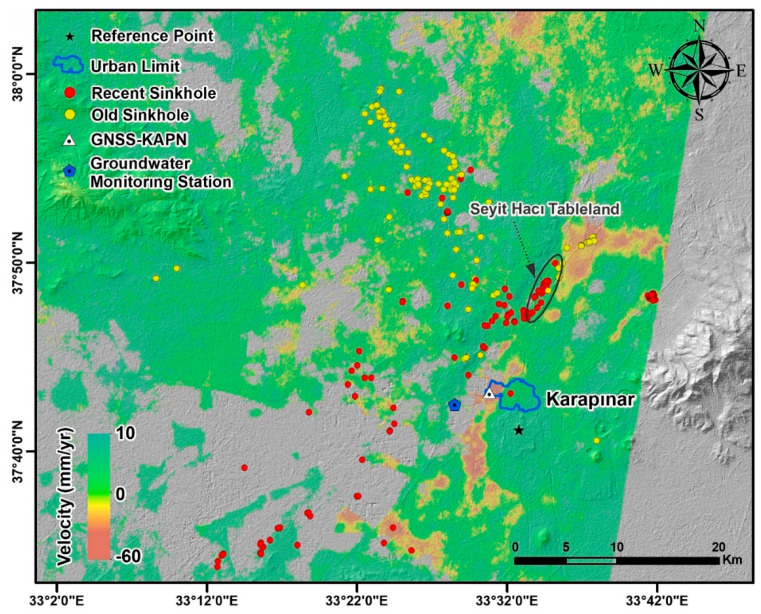
Spatial Relations between subsidence and sinkholes. Sinkhole locations overlying the Sentinel-1 velocity map of the study area. The map shows that sinkholes occur mostly in areas of no subsidence (green) or in decorrelated areas with no subsidence rate information.

The Karapınar region is subjected to both land subsidence and sinkhole activity. Our InSAR time series analysis revealed that land subsidence occurred non-homogenously in patches of variable sizes ranging from hundreds of meters to several km in width (Figure 4 and Figure 9). Sinkholes were developed in the wide area throughout the Karapınar region, but show higher concentrations along several linear features, such as the Seyit Haci Tableland (Figure 9). A comparison between sinkhole and subsidence locations reveal that most sinkholes occur in decorrelated areas (masked areas with no subsidence information) or in areas with a negligible subsidence rate (green in Figure 9). Decorrelation in the Karapınar region mainly occurred in agricultural land, due to seasonal land-use changes. Thus, sinkholes developed in agricultural land, as shown in Figure 1, cannot be detected by InSAR.

However, a few old sinkholes formed in the areas with relatively high subsidence rates near the Seyit Haci Tableland and the southern section of the study area (Figure 9). The relations between these old sinkholes that were formed before 2010 and the subsidence rate cannot be performed, as the SAR data cover a later period of 2014–2018. Sinkhole occurrence in correlated areas suggests that sinkholes occurred in areas with a negligible subsidence rate. We explain these observed relations between sinkholes and negligible subsidence by the strong cohesion of the near-surface rock layer (caprock) with a thickness of meters to tens of meters in areas prone to sinkhole occurrence (Figure 1b,e). The high cohesion caprock layer resists subsidence in response to aquifer system deformation due to groundwater level drop and, hence, allows the development of subsurface cavities due to deformation of deeper deformable rock units [56]. As subsurface cavities continue growing upward toward the surface, the caprock layer responds by either sudden collapse (Figure 1b,e) or rapid subsidence (Figure 1d) [57].

In a previous study carried out by [28,29] on the Israeli dead sea coastline, precursor deformations were determined with the InSAR technique using COSMO-SkyMED images prior to sinkhole collapse. The bare and arid lands along the Dead Sea shores had minimized temporal deceleration. Therefore, images with high coherence were obtained, and a clear precursor deformation information was detected. The Karapınar region is heavily cultivated and, hence, results in wide decorrelated areas. Furthermore, all of the sinkholes formed during the InSAR observed period (May 2014–May 2018) developed in agricultural lands. Therefore, the lack of subsidence information about the areas where the formed sinkholes are located could not be obtained (Figure 9).

## 6. Conclusions

In this study, we adopted multi-sensor and multi-temporal InSAR observations combined with GNSS and groundwater level records to investigate land subsidence and its relations to sinkhole activity and groundwater depletion in the Karapınar basin, Turkey. We processed InSAR observations acquired by COSMO-SkyMed and Sentinel-1 from October 2014 to March 2018. The InSAR results reveal that the Karapınar region experienced significant land subsidence in 1–10 km wide patches, reaching rates of 70 mm/year. For both InSAR datasets, reliable subsidence rates were obtained over bare land that yielded high interferometric coherence. However, seasonal land-use changes in the Karapınar region resulted in wide decorrelated areas, mostly over agricultural land. Land subsidence results obtained from X-band COSMO-SkyMed and C-band Sentinel-1A detected similar rates and spatial patterns of subsidence. Temporal subsidence patterns, obtained from GNSS, COSMO-SkyMed, and Sentinel-1 time series, showed a continuous subsidence with a significant seasonal component, which correlate well with the temporal pattern of groundwater level change in the region. Our study indicates a significant increase in subsidence rate from 15 mm/yr during 2002–2010 [35] to 70 mm/yr during the period of 2014-2018 (this study). The relations between sinkhole activity and subsidence indicate that most sinkholes occur in decorrelated, agricultural areas or in areas with small subsidence rates. Sinkhole occurrence in slow subsidence areas reflects the strong cohesion of near-surface rock layers that resist subsidence but yield to collapse in response to aquifer system deformation induced by groundwater extraction.

## Data Availability

Data sharing not applicable.
